# Splenic abscess as a paradoxical response to chemotherapy in tuberculous pleural effusion

**DOI:** 10.4103/1817-1737.58961

**Published:** 2010

**Authors:** Ramakant Dixit, Paras Nuwal, Manoj Arya

**Affiliations:** *Department of Tuberculosis and Respiratory Medicine, J. L. N. Medical College, Ajmer, India*; 1 *Department of Pathology, J. L. N. Medical College, Ajmer, India*

**Keywords:** Paradoxical response, splenic abscess, tuberculosis

## Abstract

We report a rare case of tuberculous pleural effusion that developed multiple splenic abscesses after antituberculosis chemotherapy. She responded to addition of oral corticosteroid to antituberculosis regimen. The relevant literature, regarding pathogenesis and clinical importance of recognizing a paradoxical response, is discussed.

Paradoxical response refers to enlargement of old lesions or appearance of unexpected new ones during apparently adequate and effective antituberculosis therapy. This has been reported to occur in cases of intracranial tuberculoma, lymph nodes, pleural and pulmonary tuberculosis.[1] However, development of splenic abscess as a paradoxical response during successful chemotherapy has not been described previously in a patient without human immunodeficiency virus (HIV) infection, although there is a recent report on spontaneous splenic rupture as manifestation of immune reconstitution inflammatory syndrome (IRIS) in an HIV type-1 infected patient with tuberculosis.[2] We report such a case in view of rarity and the need for awareness of this phenomenon.

## Case Report

A 21-year-old female presented with left-sided chest pain and low grade fever for one month. The clinical examination and X-ray chest were suggestive of moderate pleural effusion. She underwent thoracocentesis and about one liters of straw colored fluid was drained. The fluid was exudate (protein 4 gm %), lymphocytes predominant (cells 6800/mm^3^, 90% lymphocytes) with adenosine deaminase (ADA) level of 120 units. Her routine investigations including blood count, fasting blood sugar, liver function tests, renal function tests, urine examination, etc., were normal but erythrocyte sedimentation rate (ESR) was 110 mm in the first hour. Mantoux test revealed an induration of 15 mm. She was HIV sero negative and ultrasound abdomen was normal. Pleural biopsy revealed features of tubercular granulomas. Pleural fluid culture by Bactec method revealed *mycobacterium tuberculosis* sensitive to first line antituberculosis drugs.

She was initiated on intermittent, directly observed, thrice weekly antituberculosis chemotherapy with isoniazid (600 mg), rifampicin (450 mg), and pyrazinamide (1,500 mg), under category III revised national tuberculosis control program of India. She responded with above treatment and became asymptomatic with regression of pleural effusion that disappeared completely after four weeks of therapy.

**Figure 1:**
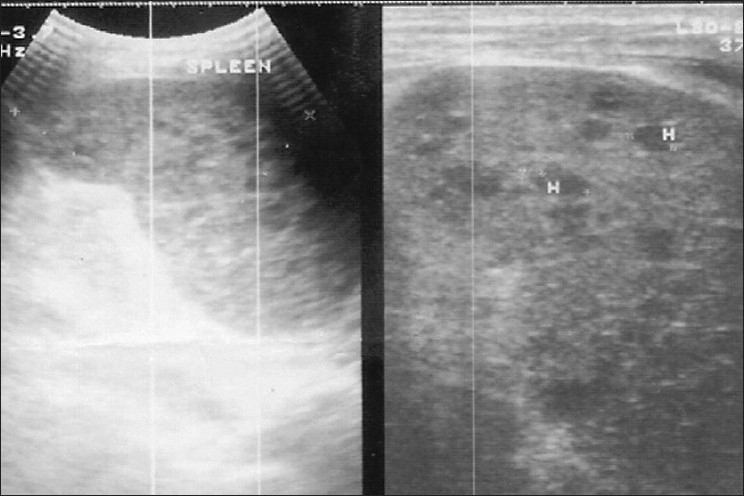
Ultrasound abdomen showing multiple hypoechoic lesions in the spleen

**Figure 2:**
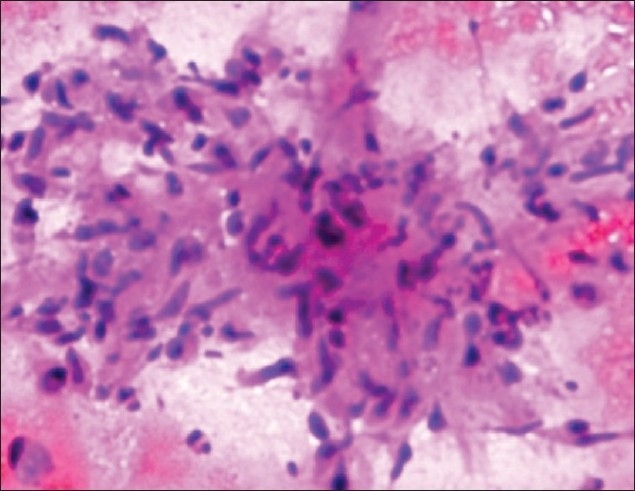
Photomicrograph of splenic abscess cytology showing epitheloid cells, lymphocytes and areas of caseous necrotic background suggestive of tuberculosis (H and E, ×200)

She remained asymptomatic till six weeks when she noticed dull aching pain abdomen at epigastrium and left hypochondrium. The pain gradually increased and did not subside with the addition of omeprazole and antacids. There was no associated vomiting and diarrhea. On examination, there was tenderness at left hypochondrium. Ultrasound abdomen this time revealed mild splenomegaly with multiple hypoechoic areas (the largest one measuring 19 × 15 × 10 mm^3^, at lower part) [Figure 1]. CT scan abdomen also confirmed enlarged spleen with multiple miliary size abscesses. There were no other intraabdominal abnormalities. Ultrasound-guided fine needle aspiration cytology (FNAC) of splenic lesion revealed the collection of inflammatory cells including epitheloid cells, lymphocytes, multinucleated giant cells with extensive caseous necrosis; but no acid-fast bacilli [Figure 2].

A possibility of paradoxical response to antituberculosis therapy was strongly considered. She was given a course of oral prednisolone starting at 40 mg/day that was rapidly tapered off in four weeks, while she was maintained on the same antituberculosis regimen. There was marked clinical response on steroid therapy and repeat ultrasound abdomen, following a course of systemic corticosteroid showed near complete resolution of the splenic lesions; which completely disappeared at the end of antituberculosis treatment that patient received for six months.

## Discussion

Tuberculous involvement of spleen is very rare and usually seen in disseminated or miliary form of the disease.[3] Many reported cases of splenic tubercular abscess are found to have underlying HIV infection also.[4] To the best of author's knowledge, tubercular involvement of spleen as paradoxical response during antituberculosis therapy in a nonHIV infected patient has not been reported previously.

Paradoxical worsening of disease, in spite of effective chemotherapy for tuberculosis, is generally regarded as immune-mediated. It has been suggested that active tuberculosis can result in immunosuppression through an altered cell-mediated response. Once active tuberculosis is under control after appropriate therapy, enhanced focal immune responses (immunological rebound) will recruit lymphocytes and macrophages at the site of these lesions, which than enlarge and become evident.[5] Hypersensitivity to the tubercular proteins released from the dying mycobacteria may be a factor as well.[6] These immunological lesions are supported by a relatively low mycobacterial culture rate and significant response after the use of corticosteroids.[7]

Our patient developed new splenic abscess after six weeks of antituberculosis therapy for left-sided pleural effusion. We propose that rapid mycobacterial killing following successful chemotherapy released mycobacterial products that stimulated the production of cytokines including TNF-α causing enhanced focal immune responses with accumulation of inflammatory exudates at the previously invisible microscopic tuberculous foci in the spleen. There was nothing to suggest infection with nontuberculous mycobacteria or drug resistance in our case and the new splenic lesions regressed completely with the same therapy.

It is important to recognize these clinically frustrating but benign paradoxical responses which usually do not require change in therapy. Clinicians should be aware of these events and at the same time consider other causes of inadequate response such as wrong diagnosis, inadequate drug regimen, multidrug resistant tuberculosis, atypical mycobacterial disease, complication of drug therapy, or the disease itself before attributing their signs and symptoms to paradoxical response. A better understanding of these events and newer developments in the field of tuberculosis, such as detection of latent tuberculosis infection by interferon gamma release assay (IGRA),[8] will definitely improve patient care in countries with high burden of the disease.
